# Risky business in Georgia's wild birds: contact rates between wild birds and backyard chickens is influenced by supplemental feed

**DOI:** 10.1017/S0950268822000851

**Published:** 2022-05-05

**Authors:** A. J. Ayala, L. K. Haas, B. M. Williams, S. S. Fink, M. J. Yabsley, S. M. Hernandez

**Affiliations:** 1 Department of Population Health, College of Veterinary Medicine, University of Georgia, 501 D.W. Brooks Drive, Athens, GA 30605, USA; 2 Daniel B. Warnell School of Forestry and Natural Resources, University of Georgia, 180 E. Green St., Athens, GA 30602, USA; 3 Southeastern Cooperative Wildlife Disease Study, 589 D.W. Brooks Drive, Athens, GA, 30602, USA

**Keywords:** Avian influenza virus, backyard chickens, contact rate, Newcastle disease virus, wild birds

## Abstract

Backyard chickens are increasingly popular, and their husbandry varies widely. How backyard chickens are housed may influence the accessibility of chicken feed and water to wild birds, and thus, the contact rates between both groups. Increased contacts have implications for pathogen transmission; for instance, Newcastle disease virus or avian influenza virus may be transmitted to and from backyard chickens from contaminated water or feed. Given this potentially increased pathogen risk to wild birds and backyard chickens, we examined which wild bird species are likely to encounter backyard chickens and their resources. We performed a supplemental feeding experiment followed by observations at three sites associated with backyard chickens in North Georgia, USA. At each site, we identified the species of wild birds that: (a) shared habitat with the chickens, (b) had a higher frequency of detection relative to other species and (c) encountered the coops. We identified 14 wild bird species that entered the coops to consume supplemental feed and were considered high-risk for pathogen transmission. Our results provide evidence that contact between wild birds and backyard chickens is frequent and more common than previously believed, which has crucial epidemiological implications for wildlife managers and backyard chicken owners.

## Introduction

The increasing popularity of backyard chicken ownership in the United States is giving rise to concerns regarding pathogen transmission between backyard chickens and wild birds. Many flock owners believe that backyard chicken rearing results in a ‘healthier’ and more ecologically responsible food source [1]. However, the sale, trade and husbandry of backyard chickens are poorly regulated, and as they are less likely to receive standard veterinary care, the data to support this perception are lacking [1]. Moreover, because backyard chickens often free-range, there is likely an increasing trend in contact with native wildlife because there are more backyard chickens that are now outdoors [2]. Increased contact results in a higher rate of ‘effective contact’, which is defined as an interaction between two hosts that leads to pathogen transmission from one to another [3].

Poultry husbandry practices range along a continuum, from densely populated, bio-contained commercial flocks to free-roaming chickens in urban centres [4]. Commercial flocks in the United States are generally managed under biosecurity protocols that seek to eliminate interactions with wild birds, reducing potential pathogen transfer from wildlife [5–7]. Backyard poultry are maintained under a variety of husbandry practices that influence their contact rates with wild birds [8], often lacking infrastructure to prevent contact with other animals [9]. As a result, contact rates between backyard chickens and wild birds may increase through shared resources [10]. For example, bird feeders, waterers, treat piles, chicken-feed troughs and faecal matter both attract and are used by multiple individuals [1, 11, 12]. As backyard chickens increase in their frequency of ownership, so too does the likelihood of effective contacts that backyard chickens may have with wild birds [1].

Previous studies of the backyard chicken–wild bird interface suggest pathogen transmission between both groups occurs more frequently for viruses such as highly pathogenic avian influenza (HPAIV) and avian-orthoavulavirus-1 (Newcastle disease virus (NDV), formerly known as avian-paramyxovirus-1) [13–16]. In many countries where backyard chickens are the primary source of protein, NDV and avian influenza virus (AIV) may be endemic, and both viruses can cause up to 100% mortality in affected poultry flocks [17, 18]. In the United States, backyard chickens have been cited as the source for three prior epizootics of virulent NDV (vNDV) in the Southwest [19]. Meanwhile, the 2014–2015 North American outbreak of H5Nx began in wild birds, before spilling over into backyard chickens and commercial poultry houses [20].

The epidemiology of NDV and AIV has been reviewed in depth [21, 22], yet they remain transboundary pathogens of emerging significance [6, 23, 24]. The field of NDV research has recently garnered a broader, interdisciplinary audience in response to increasing viral spillover and spillback events between chickens and wild birds from NDV of both velogenic (virulent) and lentogenic (low-virulent) origin [25–27]. While NDV remains a poultry-associated pathogen, recent studies suggest that most, if not all, wild bird species are susceptible [28]. More recently, Rock Pigeons (*Columba livia*), Double-crested Cormorants (*Phalacrocorax auratus*) and dabblers of the Anatidae have been proposed as reservoir hosts of this pathogen [6, 29]. The viral strain and host species both influence NDV transmission efficiency; however, direct transmission has many routes, including faecal–oral ingestion and the inhalation of infectious respiratory secretions [30].

At least 105 species of wild birds have been associated with wild-type AIV infections [31, 32]. Aquatic birds, primarily members of the Anseriformes and Charadriiformes, appear to maintain asymptomatic or mild infections and are thus considered reservoirs for AIV [32]. The spillover and spillback of AIV between wild birds and chickens may lead to co-infections within birds, facilitating viral reassortment and the subsequent emergence of highly pathogenic strains (HPAIV) within and into chickens [33].

vNDV and AIV are two of seven avian viruses that are listed as notifiable to the World Organization for Animal Health (OIE) [34]. Virulent strains of NDV and HPAIV cause acute disease among affected chickens and many species of wild birds [35, 36]. However, the ecology of these viruses remain underappreciated, specifically concerning which species of wild birds, due to both their susceptibility and probability of contact with backyard chickens, may become naturally infected with virulent strains [33, 37, 38].

Outside of estimates derived from mathematical models [39, 40], contact rates between backyard chickens and wild birds have not been measured in the field. In a previous study, we addressed the question of NDV susceptibility in four native passerines under experimental conditions in biosafety level-2 isolators [29]. However, natural contact rates between chickens and wild birds could not be accurately measured. To address this epidemiological knowledge gap, we performed systematic observations at three sites associated with backyard chickens in North Georgia, USA, where we identified and quantified the species of wild birds, both at the farm level, as well as those that entered areas that housed chickens. We also measured the contact rate for wild birds that came in contact with chickens. We identified the species of wild birds that: (a) shared habitat with the chickens, i.e. species richness, (b) had a high frequency of detection relative to other species and (c) encountered the chicken coops. Here, we define species richness as the total number of species detected, either per site (site species richness) or across all sites (mean species richness) [41]. Lastly, for the species that encountered chicken coops, we also measured the frequency or the ‘contact rate’ with which they did so.

## Methods

### Study sites

**Fig. 1.**
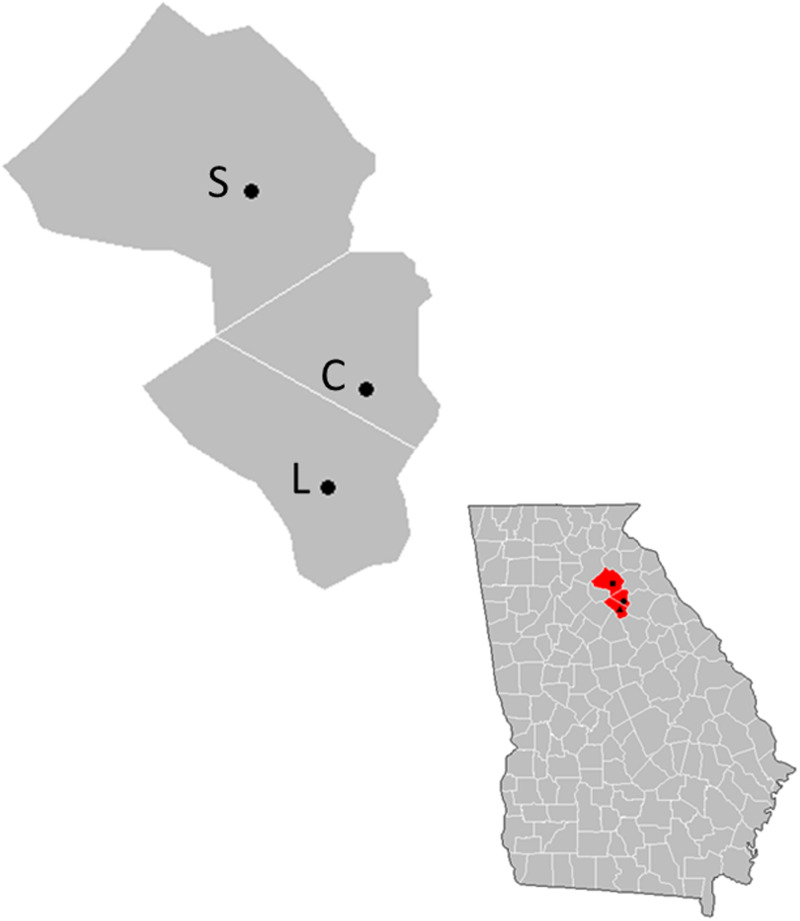
One hour-long point counts were performed from February through May 2018 at each of the three points highlighted in the upper left county map. Farm S is represented by the top point (Jackson county), Farm C is represented by the middle point (Athens-Clarke county) and Farm L is represented by the bottom point (Oconee county). The inset image (lower right) demonstrates the location of each county (in red), in North Georgia. Each farm is indicated by a point on the inset image.

We conducted observations at three backyard chicken farms in Northern Georgia (33.9519°N, 83.3576°W) USA, each of which was located in one of three counties, Athens-Clarke, Jackson and Oconee counties (Fig. 1). The three farms were of similar habitat (mixed hardwood forest surrounding open grassland) and size (average 0.004 km^2^); however, their backyard chicken husbandry methods varied (Table 1). Site L allowed all chickens to free-range during the day (*n* = 15 chickens), Site S primarily kept the chickens penned 24 h a day (*n* = 9 chickens) and Site C, which we considered our control, had an open coop, where chickens had been previously housed and subsequently removed 395 days before beginning the experiment (*n* = 0 chickens). All study sites had a wire mesh enclosure surrounding the coops, which kept out larger predators, but did not exclude most passerines smaller than American Robins (*Turdus migratorius*). To ensure that each site had avian communities that were independent of one another, all sites were located at least 10 km apart. All observations were performed between February and May 2018.

**Table 1.** Site husbandry, flock size and a summary of the number of birds observed for the three sampled sites

Site name	Flock size	Husbandry type	Observation periods^a^	Species detected^b^	Birds observed^c^	Families detected^d^	Exposed species^e^	Per cent of birds exposed^f^ (%)
Site S	*n* = 9	Cooped	20	56	706	22	12	21.4
Site L	*n* = 15	Free-range	20	46	353	20	8	17.4
Site C	*n* = 0	Control	20	52	535	23	10	19.2

a The number of observation periods refers to the number of 1 h systematic observations performed at each site throughout the field season.

b Species detected refers to the total number of species detected at each site over the course of the field season.

c Birds observed refers to the total number of individual birds observed at each site over the course of the field season, for all distance classes.

d Families detected refers to the number of families detected at each site, otherwise known as species richness.

e Exposed species indicates the total number of high-risk species out of the 14 indicated that detected in D1 and D2, at that site, over the field season.

f Per cent of species exposed indicates the percentage of the total birds detected at each site, that fell into the high-risk species category, and were detected in D1 and D2.

Twice per week, all sites were provided a commercial chicken feed mixed with supplemental foods considered highly palatable to domestic and wild species, including suet, mealworms, *Nyjer* seeds, cracked corn, sunflower seeds, and fruits and nuts [29, 42]. Supplemental feed remained in the enclosures for consumption by chickens or wild birds between visits. During the twice per week refill of supplemental feed, old or consumed food was replaced with fresh food. Based on interviews with our site owners and a review of the literature, a wide variety of non-standard, supplemental *ad-libitum* foods are provided to backyard chickens in the United States, including table scraps, fruit rinds, assorted wild bird seeds and nuts, suet, cracked corn and vegetables [12, 42, 43]. Therefore, during this experiment, we asked the owners to allow us to control and manage all feeding to reduce wild bird preference bias. This was essential to the standardisation and quantification of wild bird visits to the coops. Lastly, we also placed the supplemental feed in each coop's enclosure, as opposed to the interior of the coop, so that during observations, we could identify the species and the frequency with which they were foraging. Feeding troughs were placed no higher than 0.3 m above the ground, sanitised weekly with a 10% bleach solution to mitigate pathogen transmission, and placed within 1 m of each enclosure's opening, allowing wild birds to enter and leave freely.

### Observations of wild birds at backyard chicken sites

**Fig. 2.**
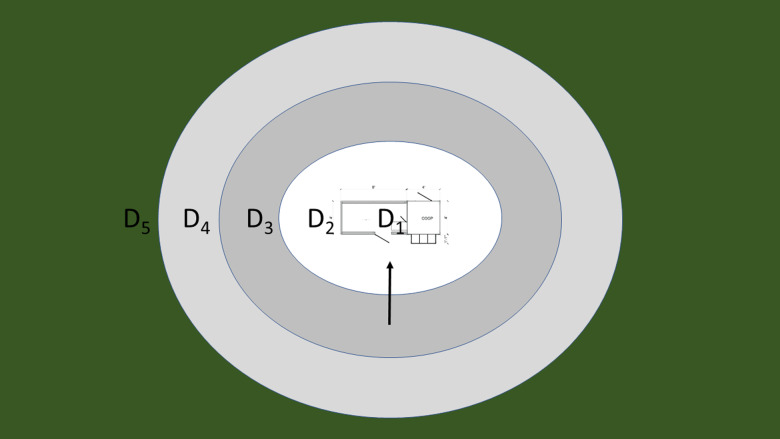
Schematic of how observations were performed at each farm. Each D_x_ corresponds to a distance class. D_1_ demarks the inside of the coop (the square on right of the coop) and wire mesh enclosure (the rectangle to the left of the coop), D_2_ correlates to the perimeter of the coop, D_3_ is 10–25 m from the coop perimeter, D_4_ is 25–50 m and D_5_ is >50 m from the coop. The black arrow denotes the observer point of view. During observations, each bird was placed into one of the five distance classes based on distance observed from the coop: (1) within the coop (distance class one, D1), (2) from the edge of the coop to <10 m (distance class two, D2), (3) between 10 and 25 m from the coop (distance class three, D3), (4) between 25 and 50 m from the coop (distance class four, D4) and (5) >50 m from the coop (including flyovers) (distance class five, D5).

To determine the frequency of detection for wild birds and contact rates between wild birds and backyard chickens, a total of 60, 1 h, unlimited-radius observations were conducted at our three sites (Table 1). Each study site was visited no more than once per day, and all three locations were visited twice per week. Observations were performed from dawn to dusk at opportunistic times to ensure that wild bird foraging behaviours were proportionately represented. Our observations were modelled upon single-observer, standard avian point count protocols in order to capture site species richness and frequency of detection [44]. However, the point count duration and the observer focus were both modified for logistical purposes. To ensure that uncommon species would also be recorded, we performed hour-long point counts, which is considerably longer than the standard 3–10 min observations [44]. In addition, we changed the observer point of view from the centre of the point count, i.e. from where the observer was standing, to face the centre of the chicken coop and enclosure, approximately 60 m away. This change ensured that birds would not avoid the coop due to observer interference (Fig. 2).

Upon arrival to each site, the observer waited for 10 min prior to beginning the census to ensure all birds returned to their natural routines. Birds were then detected both by song and by sight, using binoculars, and identified to species when possible; otherwise, unknown species were classified according to family. Once an individual was identified to species, the number of individuals was tallied according to standard avian point count protocols. Binoculars were also used when necessary to assist in identification. Point-count observers had been previously trained extensively, and then assessed to identify common Georgia, USA species [45].

Each bird noted was placed into one of five distance classes based on distance observed from the coop: (1) within the coop (distance class one, D1), (2) from the edge of the coop to < 10 m (distance class two, D2), (3) between 10 and 25 m from the coop (distance class three, D3), (4) between 25 and 50 m from the coop (distance class four, D4) and (5) > 50 m from the coop (including flyovers) (distance class five, D5) (Fig. 1). While infrequent, when individuals were observed in multiple distance classes during the same census, they were assigned to the distance class in which they spent the greatest amount of time during the 1 h observation period. These observations identified 14 high-risk species, for which we calculated the ratio at which each species was detected relative to other species, otherwise known as the frequency of detection (Supplementary Table S1), and the contact rates in which they encountered the chicken coops (Table 2). We defined a high-risk species as any bird detected in D1 during the field season.

**Table 2.** Listed in this table are the contact rates, mean minutes between each contact and mean number of contacts per day for the 14 high-risk species

Species	Site S mean exposure contact rate 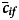	Site L mean exposure contact rate 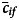	Site C mean exposure contact rate 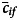	Site S mean minutes between contact *c*_*mi*_	Site L mean minutes between contacts *c*_*mi*_	Site C mean minutes between contacts *c*_*mi*_	Site S mean number of contacts per day *c*_*di*_	Site L mean number of contacts per day *c*_*di*_	Site C mean number of contacts per day *c*_*di*_
Chipping Sparrow	0.1184	0.0000	0.0000	8.4	0.0	0.0	85.3	0.0	0.0
Tufted Titmouse	0.0575	0.0102	0.0575	17.4	98.2	17.4	41.4	7.3	41.4
Northern Cardinal	0.0500	0.0142	0.0258	20	70.6	38.7	36	10.2	18.6
Myrtle Warbler	0.0217	0.0208	0.0148	46.2	48.0	67.5	15.6	15.0	10.7
Carolina Chickadee	0.0198	0.0069	0.0438	50	144.0	22.9	14.3	5.0	31.5
Mourning Dove	0.0194	0.0042	0.0000	51.4	240	0.0	14	3.0	0.0
House Finch	0.0150	0.0056	0.0000	66.7	180	0.0	10.8	4.0	0.0
Carolina Wren	0.0118	0.0000	0.0074	85	0.0	135	8.5	0.0	5.3
Blue Jay	0.0108	0.0042	0.0042	92.7	240	240	7.8	3.0	3.0
White-breasted Nuthatch	0.0095	0.0042	0.000	105	240	0.0	6.9	3.0	0.0
Song Sparrow	0.0083	0.0000	0.0083	120	0.0	120	6	0.0	6.0
Eastern Phoebe	0.0028	0.0050	0.0071	360	200	140	2	3.6	5.1
White-throated Sparrow	0.0000	0.0000	0.0500	0.0	0.0	20	0.0	0.0	36
Eastern Towhee	0.0000	0.0000	0.0050C	0.0	0.0	200	0.0	0.0	3.6

The equation results are broken down according to Sites S, L and C.

### Calculating frequency of detection and backyard chicken–wild bird contact rates

Each individual wild bird detected was tallied first according to species, then placed into one of the five distance strata (D1–D5) during each census. The frequency of detection for an individual species at each site was calculated as the total number of observations for that species, divided by the total number of wild bird observations across all sites over the field season (*n* = 1574), then multiplied by 100. For example, Tufted Titmice (*Baeolophus bicolor*) had a total of 237 detections across all sites. When divided by 1574 total detections, and multiplied by 100, this resulted in a frequency of detection of 15.06%. This was critical to ensure all species were tallied, as some species were not detected at all three sites. Across all observations, we calculated the mean detection rate for individual birds detected and the corresponding standard deviation. We also calculated the mean species richness, which is defined as the average number of species observed, in addition to calculating the mean number of families. For each high-risk species, we also calculated their overall seasonal distribution, specifically their frequency of detection in the winter *vs.* the spring season.

Although we calculated the frequency of detection for all species, we only calculated contact rates for the high-risk species which we observed in D1. Those contact rates were then calculated according to their visits to D1 and D2 throughout the field season. This approach was utilised because D1 had a clear ‘contact risk’ due to colocation with the chicken coop. In addition, D2 bordered the edges of chicken coops, thus it made the most sense to calculate contact rates for high-risk species according to their visits to D1 (in the coop) as well as D2 (which extended 10 m from the edge of the coop). In addition, NDV and AIV are transmitted both directly and through environmental sources [46]; thus, when defining a ‘contact’, each distance class was assigned a differential exposure risk. For example, species detected in both D1 and D2 throughout the field season were considered directly ‘exposed’ to the virus. Individuals detected in D3, D4 and D5 (Fig. 2) were all assigned the same exposure risk of zero and thus were not used in calculations in Equation 1.

Transmission rates are a function of a per-capita unit of time, thus we used a modified equation from Courtenay, Quinnell [47] to determine our contact rates relative to the encounters that wild birds had with backyard chicken coops. Given that we were primarily interested in interspecific interactions that would influence viral transmission, we analysed contact rates only for species that had encounters with backyard chickens and their coops, specifically for species detected within both D1 and D2.

For each observation period, the species contact rate *c*_*i*_ (Equation 1) was calculated as the sum of the individuals of a given species detected in distance class one *D*_1_ plus distance class two *D*_2_ for each observation *j*, divided by the number of minutes per observation, e.g. 60 min. The mean contact rate 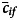 (Equation 2) of an individual species *i* at each farm *f* was then calculated as the summation of the species contact rate *c*_*i*_ (Equation 1), divided by the total number of observations *N* at each farm *f* in which species *i* was detected in any distance class to give 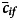. As we were using unlimited radius point counts, we did not factor observations into the numerator in which a particular species was not detected during that observation. The 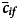 for each high-risk species at each farm are summarised in Table 2.1
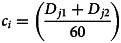
2
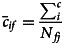


We calculated the average number of minutes *c*_*mi*_ between each contact for each species, at each site, by dividing the number one by the value for 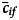 (Equation 3). Specifically, in Equation 1, the rate at which wild bird species visited the chicken coops was calculated as the mean of 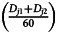
over the course of the field season. By calculating the inverse of the rate 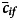, which represented the mean rate of contacts that occurred for each species *i* at each farm *f*, we obtained the average time in minutes between each species' encounter with the chicken coops [3] (Equation 3).3
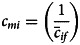


We divided the average daily activity period, i.e. 720 min, for each diurnal passerine species included in this study by the variable *c*_*mi*_ from Equation 3, which represented the mean amount of time between visits for each species *i* at each farm *f*. This provided us with the variable *c*_*di*_ in Equation 4, which represented the average number of chicken coop visits made by each high-risk species per day, at each site.4
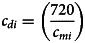


### Statistical analyses

Statistical analyses were performed using R version 3.6.1. Due to non-normalised data, we performed a Spearman's rank-order correlation to examine whether a significant relationship existed between the frequency of detection for each wild bird species, and that species' corresponding mean contact rate with the coops.

## Results

### Frequency of detection

Over 60 h of observation, 1574 individual wild birds were detected across the three sites in northern Georgia, USA, comprising 72 species from 24 families (Supplementary Table S1). The mean detection rate and standard deviation was 26.2 ± 13.5 wild birds per observation period, the species richness was 12.7, and the mean number of families was 9.3. Across all sites, Tufted Titmice (15.6%), Northern Cardinals (*Cardinalis cardinalis*) (13.2%), Carolina Chickadees (*Poecile carolinensis*) (7.0%) and Carolina Wrens (*Thryothorus ludovicianus*) (6.0%) were the four most commonly detected species, in which the percentages given here are the total percentages of all observations.

Site S, the cooped flock, had the greatest number of overall detections (44.9%), as well as the greatest species richness −77.8% of the total observed species were detected at Site S. Site L, the free-ranging flock, had the fewest number of overall detections (22.4%), as well as the lowest rank in terms of species richness, with 63.9% of the total species detected. Detections at Site C, the control site, comprised 32.7% of the 1574 observations and was the median in terms of species richness, with 70.8% of the total species observed at that site.

Site C had a higher frequency of detection for Carolina Chickadees than at Site S, where Chipping Sparrows had the highest frequency of detection. Myrtle Warblers (*Dendroica coronata*) had the highest frequency of detection at Site L. Several species, detected at Site C and S, were not detected at Site L, including Eastern Towhees (*Pipilo erythrophthalmus*), White-throated Sparrows (*Zonotrichia albicollis*) and Song Sparrows (*Melospiza melodia*).

The species with the highest frequencies of detection were more likely to be detected in D1 and D2 than in D3–D5; however, there were some exceptions. Northern Cardinals were frequently detected throughout all distance classes (D1–D5), whereas Myrtle Warblers were most commonly detected in D2 and D3.

**Fig. 3.**
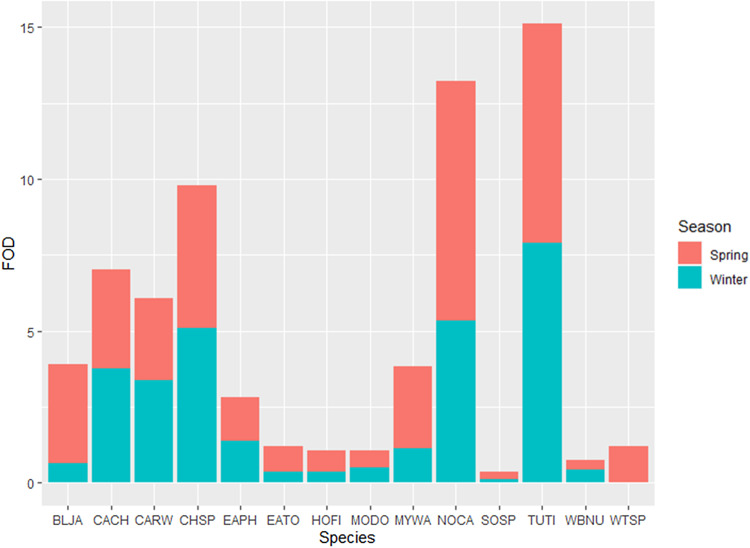
Seasonal breakdown of the frequency of detection for high-risk species. On the *y*-axis, FOD stands for the frequency of detection, and on the *x*-axis are the four-letter alpha codes for each species. The blue denotes the per cent frequency of detection in the winter, and the pink denotes the per cent frequency of detection in the spring. For each species, the alpha codes are as follows: BLJA, Blue Jay; CACH, Carolina Chickadee; CARW, Carolina Wren; CHSP, Chipping Sparrow; EAPH, Eastern Phoebe; EATO, Eastern Towhee; HOFI, House Finch; MODO, Mourning Dove; MYWA, Myrtle Warbler; NOCA, Northern Cardinal; SOSP, Song Sparrow; TUTI, Tufted Titmouse; WBNU, White-breasted Nuthatch; WTSP, White-throated Sparrow.

For some species, frequencies of detection varied by season and/or migratory behaviour (Fig. 3). Two resident species, Carolina Chickadees and Eastern Phoebes, were almost equally distributed in their frequency of detection across the winter and the spring. Other residents such as Mourning Doves, House Finches, Song Sparrows, Eastern Towhees, Northern Cardinals and Blue Jays had higher frequencies of detection in the spring than in the winter. Fewer resident species had higher frequencies of detection in the winter than the spring, such as Carolina Wrens, White-breasted Nuthatches and Tufted Titmice. Of the 14 high-risk species, only two were migratory; these were the White-throated Sparrows and Myrtle Warblers. Although both are non-breeding visitors to Georgia, both species had higher frequencies of detection in the spring than in the winter.

### Contact rates

Of the 72 total species recorded in each backyard chicken habitat, 14 species were detected in D1. Across all sites combined, these 14 high-risk species had 589 total contacts with the backyard chicken coops when accounting for visits to both D1 and D2. This comprised a contact-based frequency of detection totalling 37.4% from our 1574 detections across 60 observation hours. These species were also the most frequently detected overall, comprising 67.3% of all detections, across all distance classes.

We used contact rates to identify the species with the highest visit rates, and to extrapolate the mean number of minutes between each species' visit. With those values, we calculated a mean daily species visit rate for an average 720 min period of activity. The values for Equations 2–4 are represented in Table 2 in a species by site table. Across all sites, Tufted Titmice and Northern Cardinals had the highest backyard chicken contact rates. Species with the highest backyard chicken contact rates at Site S, C and L also included Chipping Sparrows, White-throated Sparrows and Myrtle Warblers, respectively.

### Mean daily visits

Chipping Sparrows had the highest number of visits with 85.3 mean daily visits at Site S. At Site L, Myrtle Warblers performed 15 mean daily visits, while Tufted Titmice performed 41.4 mean daily visits at Site C.

### Statistical analysis

We used a Spearman's rank-order correlation to analyse the relationship between the frequency of detection and the contact rates for all species, across all sites. The correlation test indicated a strong, positive correlation between the two variables (*ρ*_s_ (70) = 0.594, *P* < 0.0001).

## Discussion

In this experiment, we have demonstrated that a minimum of 14 wild bird species in Athens, Georgia, USA, have frequent encounters with chicken coops that may lead to pathogen exposure between backyard chickens. Although prior studies have quantified contact rates for wildlife to determine pathogen exposure [47–50], we are unaware of similar studies quantifying contact rates between backyard chickens and passerines.

Chickens spend significant periods of time in a limited area and thus it is likely that if pathogens are shed, they are concentrated within their coops and enclosures (especially for those that are not free-ranging) [50]. Our results provide evidence that contact between wild birds and backyard chickens is a common occurrence, which has important implications for pathogens shared among these species, such as NDV, HPAIV, *Salmonella* spp. and *Mycoplasma* spp. These pathogens have broad avian host ranges, may induce high mortality in affected hosts and may be transmitted via the faecal–oral route [6, 51, 52]. Our results may be extrapolated to similar species with analogous supplementary feeding habits to the high-risk families we identified in our study, including species in the Paridae, Passerellidae, Cardinalidae and Parulidae families. For instance, high contact rates between chickens in multiple backyard flocks and wild bird species with natural histories similar to Tufted Titmice, Chipping Sparrows and Northern Cardinals may be one explanation for the accelerated spread of vNDV throughout backyard chickens across the southwestern United States during previous vNDV epidemics [53–55]. Given that we did observe multiple birds consume the supplemental feed, viral particles may be transmitted through that route.

Seasonality appeared to play a role in the frequency of detection for all high-risk species apart from Carolina Chickadees and Eastern Phoebes. Due to the metabolic constraints of winter and the availability of supplemental feed to offset those constraints, we expected higher frequencies of detection for all residents during that season [56, 57]. However, only Tufted Titmice, Carolina Wrens and White-breasted Nuthatches followed the expected pattern. Surprisingly, the non-breeding migrants which overwinter in North Georgia, e.g. White-throated Sparrows and Myrtle Warblers, also had higher frequencies of detection during the spring as opposed to the winter season. While interesting, these results require further study as they may only be indicative of the seasons during which the study was conducted.

In general, our high-risk exposure species were among the most commonly detected species, and it is likely that their frequency of detection was influenced by the consistent availability of supplemental feed. It has been demonstrated that birds who consume provisional resources may undergo altered population dynamics, such as increased productivity and survival rates, and earlier breeding attempts [58, 59]. Thus, it is possible that the distributions and overall abundance of our high-risk species were inflated, in contrast to sites with similar habitats where supplemental feed was not available. Moreover, it has also been demonstrated that species who consume supplemental feed may suppress the abundance of non-supplemental feeding species [60].

The importance of these points cannot be understated as our frequencies of detection were highly correlated to our contact rates for our high-risk species. On the other hand, some low-risk wild birds were detected at frequencies that were comparable to high-risk species. For example, Red-bellied Woodpeckers (*Melanerpes carolinus*) and Pine Warblers (*Setophaga pinus*) had a 4.8% and a 2.2% frequency of detection, respectively. An additional factor that may have influenced our frequency of detection was the length of the observer period. Most point counts are conducted over a 3–10 min observation period to reduce the double-counting of birds. While measures were taken to minimise this, e.g. the use highly trained observers that could follow individual birds, the possibility remains that this metric may have some bias. What further remains uncertain is whether the frequencies of detection for low-risk species may have also been influenced by the presence of supplemental feed. Given that we did not have a coop without supplemental feed as an experimental unit, it is difficult to make an inference concerning low risk but frequently detected species.

Our results, in conjunction with the literature, suggest that the risk of spillover to these wild bird species is considerable, especially considering the high visitation rates at D1 and D2 for species such as Chipping Sparrows (Table 2). However, the timing of surveillance may play a role when sampling wild birds for exposure to viruses in which chickens serve as a reservoir, such as NDV [61]. Our prior experimental work in four wild passerines suggested that NDV shedding in these species may be sporadic, and is consistently detectable only up to 13 days [29]. Viral shedding in free-living songbirds may be under-detected, given the relatively short duration of shedding. Surveying passerines using serology also has its limitations, as songbirds have relatively short-life spans and frequently move in and out of their territories [29, 62].

Lastly, although we sought a clear answer regarding the role of husbandry in chicken–wild bird contact rates, our results may suggest that the role of husbandry as a factor in the number of daily bird visits was secondary to the role of site species richness and the availability of supplemental feed. With further speculation, our results may also suggest that the frequency with which wild bird species were detected at a site was more important than chicken husbandry when determining wild bird contact rates with chicken coops. Given that Site S accounted for almost half of the contact rates from our high-risk species, followed by Site C, it appeared that the presence of chickens in the pen had a negligible impact. However, the flock at Site L was somewhat more aggressive than the flock at Site S, which may account for the variability in contact rates and species visits not attributed to richness and the frequency of detection. On the other hand, given the lack of replicates for each treatment type (e.g. cooped, free-ranging and control), ultimately, it is difficult to make broader inferences.

In terms of management, targeted surveillance of the high-risk species we identified in this study during NDV and HPAIV outbreaks may highlight an additional mode of transmission between backyard chicken flocks that have known contact. We hope that our work will also provide additional information on how to manage epidemics of NDV in the southwest, and the current outbreak of HPAIV in the United States and Canada. For example, our results demonstrate that the removal of chickens does not deter wild birds from the enclosures where pathogens may have been present – it is the removal of freely accessible feed that would likely reduce visits, in conjunction with biosecurity aimed at preventing the wild bird visits both inside chicken houses and the perimeter. Thus, mandating the use of automatic feeders that require chickens to step on platforms to open the lids would minimise waste, and reduce the spillage that attracts wild birds and pests. In addition, the use of plastic screening over enclosures to prevent wild bird visits to backyard chicken feed may help interrupt the chain of transmission between wild birds and backyard flocks.

## Data Availability

Data relating to this study are provided in Tables 1–2, and Supplementary Table S1. The raw data for this study can be accessed by contacting the first author or the corresponding author, and will be provided to the requester within a reasonable time frame.

## Supplementary material

For supplementary material accompanying this paper visit http://doi.org/10.1017/S0950268822000851. click here to view supplementary material
