# Supplementary material for: Risky business in Georgia's wild birds: contact rates between wild birds and backyard chickens is influenced by supplemental feed
Source: Epidemiol Infect. 2022 May 5;150:e102. doi: 10.1017/S0950268822000851 (PMC9128352; doi:10.1017/S0950268822000851)
Supplement: Supplementary file 1 [file S0950268822000851sup001.docx]

**Supplemental Table 1.** Species Richness and detection ratios of all birds detected at Site L, C, and S. The species in this table were observed in the same habitats with backyard chickens across three sites in Athens, Georgia. A total of 72 species are represented in this table. Data in the column titled, “Total Detections Across all Sites’ are represented as pooled observations across all sites in order to provide comparisons for the columns with site specific detections. Rows in which the text are bolded represent our 14 high risk species.

| **Species** | **Scientific Name** | **Total Birds Detected Across all Sites** | **Site S Detections** | **Site L Detections** | **Site C Detections** | **Exposed Species** | **Detection Ratio** |
| --- | --- | --- | --- | --- | --- | --- | --- |
| Acadian Flycatcher | Empidonax virescens | 2 | 0 | 2 | 0 | No | 0.13% |
| American Crow | Corvus brachyrhynchos | 34 | 16 | 5 | 13 | No | 2.16% |
| American Goldfinch | Spinus tristis | 11 | 2 | 3 | 6 | No | 0.70% |
| American Robin | Turdus migratorius | 6 | 3 | 2 | 1 | No | 0.38% |
| Barn Swallow | Hirundo rustica | 1 | 0 | 0 | 0 | No | 0.06% |
| Black-and-white Warbler | Mniotilta varia | 10 | 4 | 4 | 2 | No | 0.64% |
| Blue-gray Gnatcatcher | Polioptila caerulea | 15 | 1 | 1 | 13 | No | 0.95% |
| Brown-headed Cowbird | Molothrus ater | 4 | 2 | 0 | 2 | No | 0.25% |
| Brown-headed Nuthatch | Sitta pusilla | 12 | 3 | 7 | 2 | No | 0.76% |
| **Blue Jay** | **Cyanocitta cristata** | **62** | **36** | **20** | **6** | **Yes** | **3.93%** |
| Blackpoll Warbler | Setophaga striata | 1 | 0 | 1 | 0 | No | 0.06% |
| Black Vulture | Coragyps atratus | 5 | 4 | 0 | 1 | No | 0.32% |
| Brown Thrasher | Toxostoma rufum | 12 | 12 | 0 | 0 | No | 0.76% |
| Black-throated Blue Warbler | Setophaga caerulescens | 6 | 3 | 2 | 1 | No | 0.38% |
| Black-throated Green Warbler | Setophaga virens | 1 | 1 | 0 | 0 | No | 0.06% |
| Broad-winged Hawk | Buteo platypterus | 4 | 0 | 0 | 4 | No | 0.25% |
| **Carolina Chickadee** | **Poecile carolinensis** | **110** | **34** | **19** | **57** | **Yes** | **6.99%** |
| Canada Goose | Branta  Canadensis | 1 | 0 | 0 | 1 | No | 0.06% |
| Cape May Warbler | Setophaga tigrine | 5 | 3 | 1 | 1 | No | 0.32% |
| **Carolina Wren** | **Thryothorus ludovicianus** | **94** | **30** | **25** | **39** | **Yes** | **5.97%** |
| Cedar Waxwing | Bombycilla cedrorum | 10 | 0 | 10 | 0 | No | 0.64% |
| **Chipping Sparrow** | **Spizella passerina** | **156** | **152** | **2** | **2** | **Yes** | **9.91%** |
| Chimney Swift | Chaetura pelagica | 7 | 4 | 0 | 3 | No | 0.45% |
| Common Grackle | Quiscalus quiscula | 7 | 7 | 0 | 0 | No | 0.45% |
| Common Yellowthroat | Geothlypis trichas | 2 | 1 | 0 | 1 | No | 0.13% |
| Dark-eyed Junco | Junco hyemalis | 3 | 3 | 0 | 0 | No | 0.19% |
| Downy Woodpecker | Picoides pubescens | 25 | 5 | 11 | 9 | No | 1.59% |
| Eastern Bluebird | Sialia sialis | 10 | 6 | 3 | 1 | No | 0.64% |
| **Eastern Phoebe** | **Sayornis phoebe** | **43** | **9** | **16** | **18** | **Yes** | **2.73%** |
| **Eastern Towhee** | **Pipilo erythrophthalmus** | **19** | **1** | **0** | **15** | **Yes** | **1.21%** |
| Eastern Wood-Pewee | Contopus virens | 3 | 2 | 0 | 1 | No | 0.19% |
| Fish Crow | Corvus ossifragus | 16 | 11 | 0 | 5 | No | 1.02% |
| Great-crested Flycatcher | Myiarchus crinitus | 10 | 6 | 2 | 2 | No | 0.64% |
| Golden-crowned Kinglet | Regulus satrapa | 6 | 2 | 1 | 3 | No | 0.38% |
| Gray Catbird | Dumetella carolinensis | 3 | 1 | 0 | 2 | No | 0.19% |
| Hairy Woodpecker | Leuconotopicus villosus | 3 | 1 | 2 | 0 | No | 0.19% |
| Hermit Thrush | Catharus guttatus | 1 | 0 | 0 | 1 | No | 0.06% |
| **House Finch** | **Haemorhous mexicanus** | **17** | **13** | **4** | **0** | **Yes** | **1.08%** |
| Hooded Warbler | Setophaga citrina | 7 | 0 | 1 | 6 | No | 0.45% |
| Indigo Bunting | Passerina cyanea | 8 | 8 | 0 | 0 | No | 0.51% |
| Louisiana Waterthrush | Parkesia motacilla | 1 | 0 | 1 | 0 | No | 0.06% |
| Mississippi Kite | Ictinia mississippiensis | 1 | 1 | 0 | 0 | No | 0.06% |
| **Mourning Dove** | **Zenaida macroura** | **17** | **8** | **8** | **1** | **Yes** | **1.08%** |
| **Myrtle Warbler** | **Setophaga coronata** | **60** | **17** | **24** | **19** | **Yes** | **3.81%** |
| **Northern Cardinal** | **Cardinalis cardinalis** | **207** | **94** | **47** | **66** | **Yes** | **13.15%** |
| Northern Mockingbird | Mimus polyglottos | 9 | 6 | 0 | 3 | No | 0.57% |
| Northern Parula | Setophaga Americana | 8 | 1 | 3 | 4 | No | 0.51% |
| Northern Rough-winged Swallow | Stelgidopteryx serripennis | 2 | 0 | 1 | 1 | No | 0.13% |
| Ovenbird | Seiurus aurocapilla | 4 | 2 | 1 | 1 | No | 0.25% |
| Pine Warbler | Setophaga pinus | 35 | 17 | 12 | 6 | No | 2.22% |
| Pileated Woodpecker | Dryocopus pileatus | 1 | 0 | 1 | 0 | No | 0.06% |
| Prairie Warbler | Setophaga discolor | 2 | 1 | 1 | 0 | No | 0.13% |
| Prothonotary Warbler | Protonotaria citrea | 2 | 0 | 0 | 2 | No | 0.13% |
| Red-bellied Woodpecker | Melanerpes carolinus | 76 | 26 | 19 | 31 | No | 4.83% |
| Ruby-crowned Kinglet | Regulus calendula | 30 | 14 | 5 | 11 | No | 1.91% |
| Red-eyed Vireo | Vireo olivaceus | 6 | 3 | 1 | 2 | No | 0.38% |
| Red-shouldered Hawk | Buteo lineatus | 5 | 0 | 1 | 2 | No | 0.32% |
| Red-tailed Hawk | Buteo jamaicensis | 1 | 1 | 0 | 0 | No | 0.06% |
| Ruby-throated Hummingbird | Archilochus colubris | 6 | 1 | 3 | 2 | No | 0.38% |
| Scarlet Tanager | Piranga olivacea | 3 | 1 | 2 | 0 | No | 0.19% |
| **Song Sparrow** | **Melospiza melodia** | **6** | **4** | **0** | **2** | **Yes** | **0.38%** |
| Summer Tanager | Piranga rubra | 1 | 1 | 0 | 0 | No | 0.06% |
| **Tufted Titmouse** | **Baeolophus bicolor** | **237** | **93** | **51** | **93** | **Yes** | **15.06%** |
| Turkey Vulture | Cathartes aura | 33 | 10 | 5 | 18 | No | 2.09% |
| **White-breasted Nuthatch** | **Sitta carolinensis** | **12** | **8** | **4** | **0** | **Yes** | **0.76%** |
| White-eyed Vireo | Vireo griseus | 4 | 0 | 0 | 4 | No | 0.25% |
| **White-throated Sparrow** | **Zonotrichia albicollis** | **19** | **0** | **0** | **19** | **Yes** | **1.21%** |
| Wood Thrush | Hylocichla mustelina | 2 | 0 | 1 | 1 | No | 0.13% |
| Yellow-bellied Sapsucker | Sphyrapicus varius | 8 | 4 | 1 | 3 | No | 0.51% |
| Yellow-shafted Flicker | Colaptes auratus | 12 | 1 | 10 | 1 | No | 0.76% |
| Yellow-throated Vireo | Vireo flavifrons | 1 | 0 | 1 | 0 | No | 0.06% |
| Yellow-throated Warbler | Setophaga dominica | 1 | 1 | 0 | 0 | No | 0.06% |
